# Unravelling the ties that bind: The intersection of obesity, osteoarthritis, and inflammatory pathways with emphasis on glucagon‐like peptide‐1 agonists

**DOI:** 10.1111/cob.12700

**Published:** 2024-08-16

**Authors:** Naadir Jamal, William Hollabaugh, Leon Scott, Sahar Takkouche

**Affiliations:** ^1^ Vanderbilt University School of Medicine Nashville Tennessee USA; ^2^ Department of Orthopaedic Surgery, Division of Sports Medicine Vanderbilt University Medical Center Nashville Tennessee USA; ^3^ Department of Medicine, Division of Diabetes, Endocrinology and Metabolism Vanderbilt University Medical Center Nashville Tennessee USA

**Keywords:** glucagon‐like peptide‐1 receptor agonist, glucose‐dependent insulinotropic polypeptide, inflammatory pathways, liraglutide, obesity, osteoarthritis

## Abstract

This narrative review article explores the complex interplay between obesity, osteoarthritis, and their associated inflammatory cascades, offering a deeper understanding of the underlying of mechanisms of inflammation and potential therapeutic interventions targeting both diseases. Through examination of the shared inflammatory pathway of obesity and osteoarthritis, our objective is to directly elucidate the relationship between these two conditions, highlighting the promising role of glucagon‐like peptide‐1 agonists in modulating inflammation and its therapeutic implications for patients with obesity and osteoarthritis.

## Graphical abstract

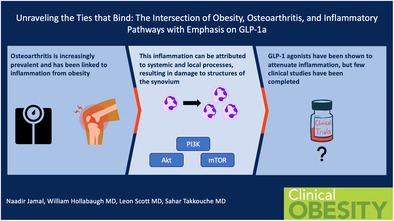

## 1 INTRODUCTION

Obesity is a global epidemic, and a major cause of morbidity and mortality worldwide. Osteoarthritis (OA), the progressive degenerative change of joints involving loss of cartilage and accompanying changes in bone and synovium, is rising in incidence.^1^ Obesity has been identified as the greatest modifiable risk factor for development of symptomatic OA, primarily due to its association with an increased mechanical risk for degenerative change in weightbearing joints.^2^ OA afflicts 10%–12% of the population, resulting in a substantial financial burden on the global healthcare system.^1^ Arthroplasty, specifically total arthroplasty, is the definitive treatment for OA in many large joints. The number of arthroplasties has doubled in the United States of America (USA) over the past decade with over 1 million performed annually, with an estimated total annual cost of $15 billion.^1^

Excess adipose tissue in the obese state was previously thought to serve as a static storage reservoir for energy. However, recent evidence suggests that adipose tissue is extremely active in intercellular signalling, releasing adipokines as signalling molecules.^3^ Certain adipokines, i.e., leptin and adiponectin, have been linked to both the propagation of obesity and development of obesity‐related complications.^3^, ^4^ Moreover, obesity and its associated metabolic disturbances, i.e., insulin resistance and hyperlipidemia, create a pro‐inflammatory environment.^5^ Over the last two decades, the role of inflammation has been progressively described in OA, which was originally regarded as a disease of “wear and tear”.^6^ Rather, the pathophysiology of OA also involves inflammatory damage to the joint, which may be exacerbated by the pro‐inflammatory environment created by obesity. With the growing evidence supporting the role of inflammation in obesity as a component of the mechanism for development and propagation of OA, it is crucial for clinicians to understand the inflammatory mechanisms driving these diseases. This understanding is necessary to provide the highest level of comprehensive care for patients.

In this review, we first describe treatment concepts for obesity and OA, and the inflammatory pathways connecting these two conditions. Next, we discuss how obesity can modulate OA, specifically how metabolic and inflammatory derangements associated with obesity may exacerbate degenerative joint pathology. We next describe why glucagon‐like peptide‐1 (GLP‐1), a hormone known for its effects on weight and metabolism, has garnered interest as a therapeutic agent due to its role in modulating inflammatory pathways. We also highlight the limited prior appraisal of glucagon‐like peptide‐1 receptor agonists (GLP‐1a) in the context of OA. Lastly, we discuss the potential therapeutic roles of GLP‐1a and dual agonists of GLP‐1 through the lens of contemporary pre‐clinical and clinical studies. The aim of this review article was to examine how obesity impacts the biochemistry and inflammatory cascades in joint tissues, particularly in the context of OA, and to evaluate novel pharmacotherapies used for treating obesity, considering their potential dual role in both treating and reducing chronic inflammatory disease states.

## 2 TREATMENT CONCEPTS FOR OSTEOARTHRITIS AND OBESITY

Non‐operative multidisciplinary weight loss interventions using diet modifications, counselling, and exercise programs demonstrate a positive impact on weight loss over time.^7^ Operative weight loss interventions, i.e., metabolic bariatric surgery (MBS), present another option for patients with obesity and have shown excellent long‐term results. The two most performed MBS procedures in the USA are Roux‐en‐Y gastric bypass (RYGB) and vertical sleeve gastrectomy (VSG). Patients undergoing RYGB have lost 27.5% of their baseline weight, with VSG not far behind at 17.8%.^8^ Moreover, only 3.4% of patients who underwent RYGB regained weight to within 5% of their baseline at the 10‐year mark.^8^ While the beneficial effects of MBS were thought to be due to intake restriction and malabsorption, evidence has also linked changes in neurohormonal signalling to positive outcomes. For example, postprandial GLP‐1 levels are increased 10‐fold following RYGB and VSG.^9^ Additionally, the approval of GLP‐1a for weight loss has changed the landscape of options available to patients, as they produce the neurohormonal signalling changes that constitute part of the beneficial effect of MBS.^10^ While studies show that they independently induce a lower magnitude of weight loss than MBS, GLP‐1a are now appealing options for patients not interested in surgery.^11^

Moreover, obesity is a significant risk factor for the development and progression of OA.^12^ Given this relationship, treatment recommendations for OA include weight management, such as the American Academy of Orthopaedic Surgery, who have guidelines supporting non‐pharmacological solutions for treatment of OA, including weight loss for individuals who are overweight.^12^ Studies focusing on weight loss interventions in knee OA show improvements in pain, function and a reduction in the rate of knee arthoplasty.^13^, ^14^ While the initial understanding of the pathophysiology of OA focused on the idea of excess mechanical load driving degenerative changes in articular cartilage, bone, and synovium defining the disease, the association between obesity and knee OA is more complex than the relationship between weight and pressure inside a joint. Extracellular matrix biomarkers (ECM), i.e., collagen‐2 and fibulin‐3, have been used to track cartilage degeneration, and studies have displayed that weight loss alone does not stop damage to joints as measured by these biomarkers.^15^ This example indicates that mass reduction from weight loss alone is not sufficient to curtail the progression of OA.

## 3 INFLAMMATORY PATHWAYS IN OBESITY AND OSTEOARTHRITIS

Adipose tissue is an active endocrine organ that secretes signalling molecules which mediates the interplay between adipocytes, nerves, and immune cells. Recently, studies have uncovered how obesity leads to a pathologic state of inflammation that may predispose and/or exacerbate numerous diseases, including OA.^16^ Mechanistically, the accumulation of excess fat in adipocytes leads to downstream signalling effects of adipocyte death, hypoxia, and mechanotransduction between local cells and the ECM that provokes an inflammatory response.^16^ This response leads to derangements in signalling molecules including cytokines and adipokines, further recruiting and activating immune cells.^16^

In obesity, the overall pro‐inflammatory phenotype is driven not only by the increase in total fat mass, but also by the ratio of types of fat present. Notably, the proportion of white adipose tissue is increased in obesity compared to subjects in the normal BMI range.^16^ White adipose tissue is responsible for cytokine release and production of adipokines. Many of these signalling molecules, namely the cytokines IL‐1, IL‐6, IL‐15, IL‐18, and TNF‐a, in addition to the adipokines adiponectin, leptin, resistin, and visfatin, have been identified as inflammatory mediators in the pathogenesis of OA.^17^ Subsequent changes to the activity levels of macrophages, neutrophils, and lymphocytes also leads to an overall pro‐inflammatory phenotype.

Both the quality and quantity of immune cells trend towards an overall pro‐inflammatory profile in the obese state. Though both neutrophils and macrophages are activated through interactions with adipocytes, the first cellular responders recruited by stressed adipocytes are neutrophils, the primary executors of the acute inflammatory process.^18^ In epidemiological studies, obese individuals have exhibited higher circulating leukocyte counts, and in particular neutrophils, compared to lean individuals.^19^ In response to adipocyte signalling, activated neutrophils release inflammatory mediators that attract macrophages and innate immune cells. In OA, neutrophils are abundant in the synovial fluid, and production of neutrophil elastase and other proteolytic enzymes by activated neutrophils, is correlated with cartilaginous damage and radiographic progression of disease, with neutrophil elastase shown to directly induce chondrocyte apoptosis.^20^, ^21^ The observed shift towards a pro‐inflammatory profile in obesity also occurs in macrophages, which are the most abundant resident immune cells in adipose tissue. Macrophage populations in states of obesity are shifted from M2 to M1 phenotypes.^22^ While M2 macrophages generate anti‐inflammatory molecules like IL‐10 and TGF‐B, M1 macrophages produce cytokines like IL‐6, a potent eosinophil stimulator, and TNF‐a, a pro‐inflammatory cytokine.^22^ These cytokines are established mediators of OA.

Though less described than innate cell dysfunction, knowledge of adaptive cell dysfunction in obesity is the subject of recent studies and offers a similar picture. B lymphocytes, like macrophages, reside in all types of adipose tissue and show similar phenotypic changes in obesity. A high‐fat diet mouse model showed infiltration of B cells into visceral adipose tissue, with these cell populations showing increased proportions of IgD‐ IgM+ cells and higher rates of class switching to pro‐inflammatory IgG+ subtypes, a picture consistent with active immune response.^23^ However, in OA, B‐cells make up a small portion of infiltrating immune cells in the synovium, and evidence for a potential role in OA has been slow to emerge.^24^ Although, a recent study showed that circulating B cells from patients with OA identify auto‐antibodies targeted to proteins in the synovial fluid.^25^ Likewise, T‐cells display a quantitative phenotypical change in response to obesity. Populations of both CD4+ and CD8+ T cells increase in obesity, with shifts that result in increases in proportions of inflammatory TH1 cells and cytotoxic T cells, as well as reductions in regulatory T cells that regulate inflammation.^26^ The shift away from TH2 cells to TH1 cells propagates the pro‐inflammatory changes in macrophage profile, as cytokines from TH2 cells lead to the production of the M2 subtype of macrophage.^26^

As described, immune cell mediators of inflammation likely perpetuate the degenerative changes in osteoarthritic joints, supporting the idea that OA is an inflammatory condition, not just a process of “wear and tear.” This cycle occurs in all tissue types that make up the joint, but most prominently occurs in the synovium, the critical layer of connective tissue that lines the articular capsule and lubricates the joint interface. Synovitis has been observed in patients prior to onset of radiographic evidence of OA, indicating inflammation is present early in the disease course.^27^ The product is a cycle of chronic inflammation, tissue damage, and structural remodelling that leads to progressive loss of function of the joint.

## 4 OBESITY AS A DRIVER OF OSTEOARTHRITIS

**FIGURE 1:**
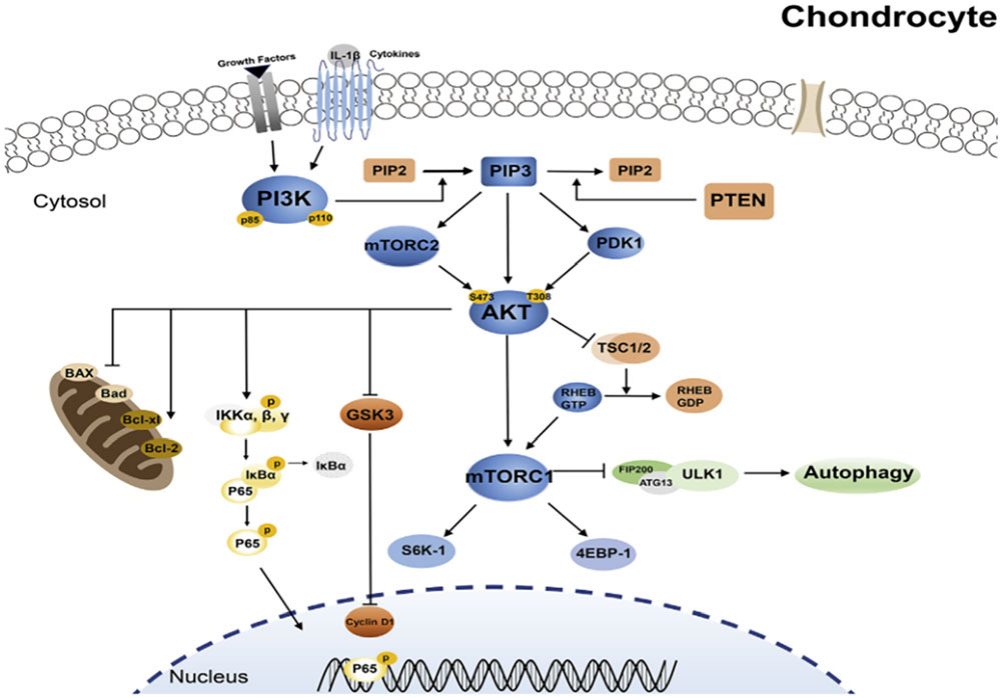
Abbreviated scheme of the PI3K/AKT/mTOR signalling pathways in a chondrocyte.

**FIGURE 2:**
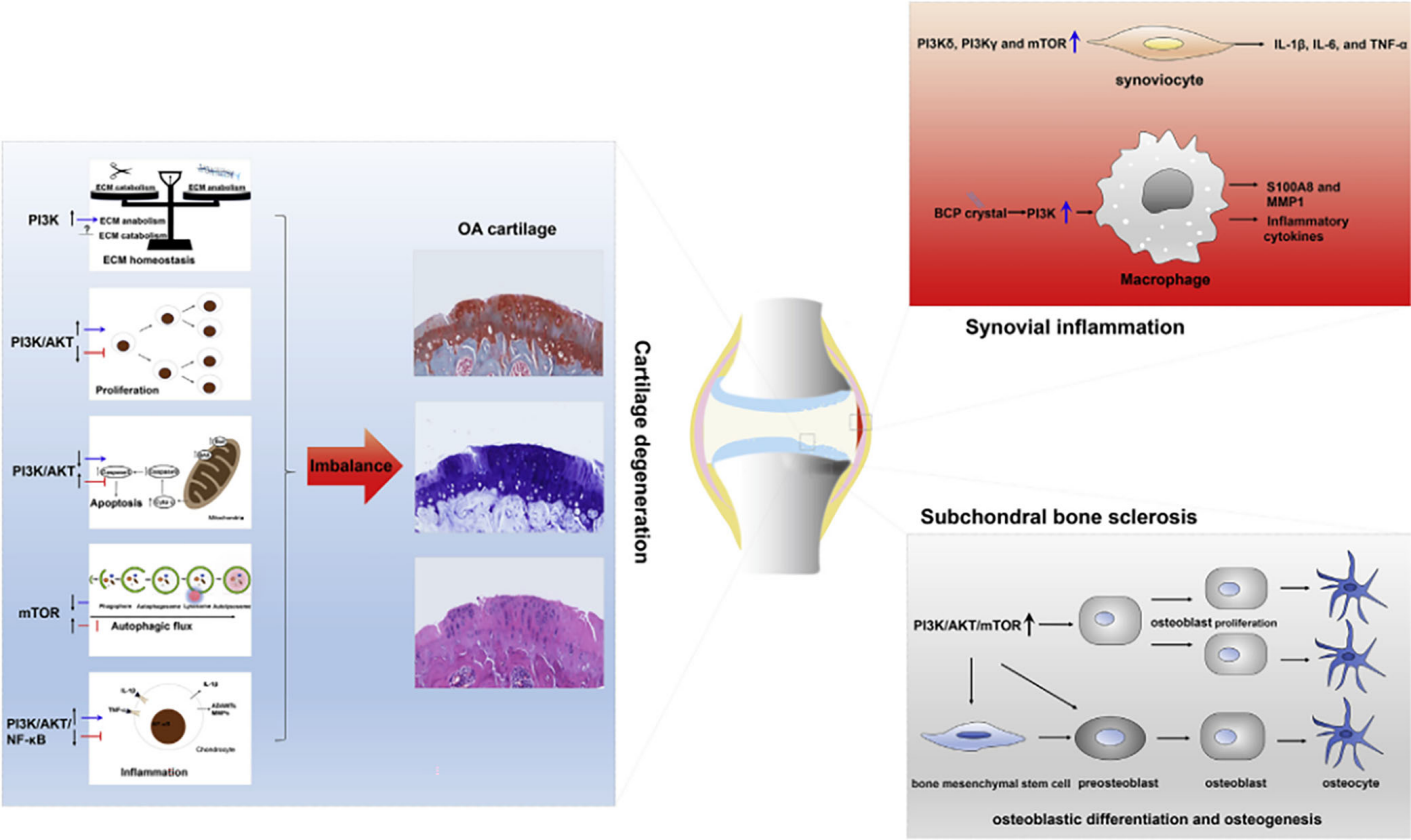
Simplified scheme displaying how a pro‐inflammatory imbalance of the PI3K/AKT/mTOR pathways contributes to OA progression, particularly synovial inflammation, and subchondral bone sclerosis.

Obesity modulates OA by leading to metabolic derangements that may exacerbate degenerative joint pathology. At the molecular level, dysregulation of phosphoinositide 3‐kinases (PI3K)/AKT signalling pathways, a critical cascade involved in both metabolism of cell and immune response, is implicated in both obesity and OA (Figure 1). In states of chronic excess caloric intake, increases in circulating free fatty acids leads to deleterious effect on metabolic function in the liver, pancreas, and other insulin‐sensitive tissues, culminating in impaired PI3K/AKT signalling that propagates inflammatory and metabolic abnormalities.^28^ Critically, dysfunction of this pathway is an essential part of the pathogenesis of OA, leading to alterations in ECM homeostasis, chondrocyte proliferation, autophagy and apoptosis, and specifically synovial inflammation and subchondral bone sclerosis of OA (Figure 2).^29^ For example, upregulation of PI3K subtypes prompts release of IL‐6 and TNF‐a from synovicocytes and induces macrophages to release MMP1, contributing to synovial inflammation (Figure 2).^30^ Furthermore, activation of PI3K, AKT, and mTOR drives differentiation of bone mesenchymal stem cells and promotes osteoblast proliferation, driving the process of subchondral bone sclerosis.^31^

Adipokine production, i.e., leptin, adiponectin, resistin and visfatin, is a key mechanism through which obesity leads to a pro‐inflammatory state. The most described adipokine, leptin, is directly implicated in obesity. In joints, leptin upregulates receptors for pro‐inflammatory cytokines, i.e., IL‐6 and IL‐8, in synovial fibroblasts.^4^ The expression of both leptin and adiponectin receptors have been identified on chondrocytes and subchondral osteoblasts, and subchondral osteoblasts in osteoarthritis overproduce leptin compared to normal cells.^32^ Additionally, leptin induces expression of matrix metalloproteases (MMP) ‐2 and ‐9, potent catabolic agents that remodel the architecture of the ECM inside the joint.^33^ Critically, downstream effects of leptin, such as inhibition of white adipose tissue lipogenesis, have been directly attributed to PI3K signalling, underlining the central role of this pathway.^34^ Additionally, adiponectin's involvement in degenerative joint disease is thought to involve cartilage matrix degradation due to positive associations with circulating levels of cartilage oligomeric protein and increased MMP‐3 in the joint.^35^ Moreover, resistin has produced OA‐like pathology when administered intra‐articular in mouse models, leading to leukocytic infiltration of the synovium and synovial hypertrophy.^36^ In clinical studies, there is also a positive association between serum resistin levels and cartilage defects.^37^

While obesity creates a state of low‐grade systemic inflammation, localized joint inflammation also plays an important role in OA. For example, in knee synovial fluid samples of patients with obesity and OA, levels of adiponectin, leptin, resistin, and visfatin were found to be elevated.^38^ Moreover, the infrapatellar fat pad (IFP), which is a site of adipose tissue adjacent to the synovium of the knee that increases in volume in states of obesity, has recently been implicated in biochemical pathways of inflammation.^39^, ^40^ In mouse models of OA, the IFP showed increased volume, adipocyte size, and vascularity and a positive association with osteophyte size.^39^ Another study also showed that the IFP in OA‐models had elevated inflammatory cell subtypes and markers, suggesting a local role in inflammation.^40^ Further, the IFP has been found to have a higher concentrations of inflammatory signalling molecules when compared to subcutaneous adipose tissue.^41^

The pro‐inflammatory effects in the IFP may mirror phenotypic changes in adjacent synovial cells that may propagate the inflammatory state. For example, one study showed that IL‐6 produced by the IFP influences synovial fibrosis through activation of synovial fibroblasts, a typical histologic feature of OA.^42^ In another study, incubation with IFP adipocytes led synovial fibroblasts to produce IL‐6, IL‐8, MMP‐1, MMP‐2, and MMP‐3, potent inflammatory and catabolic factors.^43^ Notably, this was not observed when the fibroblasts were incubated with subcutaneous adipose tissue. The association between the IFP and inflammation was also identified in human studies. In a study of patients with knee OA, inflammation of the IFP, as defined by findings on MRI, was associated with increased pain and radiographic cartilage defects.^44^

Inflammation resulting from obesity is not only associated with the molecular biology of OA but also the accompanying symptoms of OA. This association is anticipated, given the fundamental intertwining of inflammation and pain. Numerous cytokines discussed above are involved in both the initiation and persistence of pain through direct activation of nociceptors in the joint.^45^ Clinical studies have also shown that pain is associated with levels of intraarticular adipokines, often exhibiting joint‐specific differences.^45^

In addition to the changes associated with increases in overall fat volume, it is important to recognize the role of diseases associated with obesity that may amplify the inflammatory state generated by obesity, thereby potentially advancing the pathology of OA. For example, dyslipidemia has been linked to joint inflammation, with one meta‐analysis reporting that the risk of dyslipidemia in patients with OA is significantly higher than in those without OA.^46^ Also, atherosclerosis is posited to jeopardize blood supply to the joint, limiting the ability to heal structural changes from inflammatory and/or mechanical insults. This theory is supported by studies showing a strong association between OA and atherosclerosis.^47^ Moreover, metabolic syndrome has also been linked to pro‐inflammatory changes. One mechanism through which this occurs is through increased activation of macrophages, immune cells that are critical to the inflammatory processes that characterize degenerative joint damage in OA.^5^ Furthermore, metabolic syndrome has been hypothesized to exert direct pathologic effects on chondrocytes, contributing to the development of OA, via production of pro‐inflammatory and catabolic mediators, suppression of autophagy and by promotion of cellular senescence.^48^

## 5 GLP‐1: A POTENTIAL THERAPEUTIC TARGET

GLP‐1, a hormone primarily released from pancreatic alpha‐islet cells and enteroendocrine cells in the gastrointestinal tract, is a key modulator of energy balance and insulin regulation. Initial understanding of its functions centered around metabolic effects, such as its role in regulating blood sugar levels, decreasing gastric emptying and in appetite regulation.^49^ The manipulation of these functions through use of GLP‐1a medications in patients with obesity has resulted in substantial weight loss.^50^ Because of this effect, the prescription of GLP‐1a medications as a weight loss tool has rapidly increased over the past decade.^50^

Studies have displayed that GLP‐1 is much more than an insulin secretagogue and metabolic regulator; it is a potent regulator of immune cells and inflammatory pathways. For example, recent studies demonstrated that GLP‐1 signalling triggered pathways involved in B lymphocyte growth and differentiation.^51^ Encouraging for the treatment of OA, pharmacotherapy using GLP‐1 agonists has not only led to significant weight loss, but also a reduction in the inflammation associated with obesity. Specifically, a randomized control trial (RCT) conducted in adults with obesity who received a combination of exercise and liraglutide daily showed a 6.1% reduction in abdominal fat percentage and a 43% reduction in high‐sensitivity C‐reactive protein, a highly sensitive marker of general inflammation in the body.^52^

This paradigm shift inspired investigation into the exact mechanisms through which this occurs in various inflammatory pathologies, including OA. In chondrocytes, GLP‐1 receptor activation was linked to dampening of chondrocyte apoptosis in addition to an overall anti‐inflammatory effect.^53^ In pancreatic islet cells, GLP‐1 was found to modulate apoptosis through both the PI3K and PKA pathways, prompting investigation as to whether this association also occurs in the pathophysiology of OA.^54^ Moreover, GLP‐1 displayed protective effects against the effects of IL‐1B and triglycerides, which act as potent inducers of endoplasmic reticulum stress and apoptosis in intraarticular cells.^53^ However, when the PI3K/AKT pathway was knocked out, these protective characteristics of GLP‐1 were lost. Likewise, in chondrocytes that were treated with triglycerides to induce inflammation, GLP‐1 was found to dampen the nFkB pathway, reduce expression of pro‐inflammatory cytokines, and lessen catabolism of the ECM, the integrity of which is critical to preventing apoptosis.^53^

These findings extend to in vitro studies, where an experimental group of mice underwent resection of the ACL and meniscus to induce OA‐type pathology, followed by treatment with liraglutide, a GLP‐1a medication. Subsequent histology revealed significantly reduced cartilage degradation and severity of OA.^53^ In another mouse model, OA‐type pathology was caused using injection of monoiodoacetate (MIA), a widely used method for OA induction. Subsequent assays of chondrocytes revealed downregulation of the GLP‐1 receptor in subjects with induced joint inflammation.^55^ Furthermore, in this study, inflammatory mediators were found to be upregulated, generating the hypothesis that GLP‐1 is involved in key steps of the inflammatory pathways of OA. Overall, pharmacotherapy with GLP‐1a displayed therapeutic promise through anti‐inflammatory effects in in vitro models.

Few prospective in vivo trials have emerged focusing on evaluating GLP‐1 as a pharmacotherapy for OA. In contrast to rheumatoid arthritis, where disease modifying drugs and biologics can modify the course of disease and prevent disease progression, disease‐modifying pharmacotherapy options for OA have remained stagnant.^56^ While non‐steroidal anti‐inflammatory drugs and corticosteroids (e.g., oral or by intramuscular or intraarticular injection) may provide symptomatic relief, they have not been shown to slow the progression of OA.^56^ This leaves patients with refractory OA with no choice but to consider more advanced treatments, including arthroplasty. The prospect of GLP‐1a serving as a disease‐modifying pharmacotherapy for OA could be significant.

Liraglutide has demonstrated favourable outcomes in initial basic science investigations. In a mouse model, intraarticular MIA was used to induce an OA‐like state, and the animals were administered various doses of liraglutide.^57^ Results suggested liraglutide caused a dose‐dependent decrease in inflammatory mediators, particularly IL‐6, PGE2, and nitric oxide.^57^ Similarly, there were corresponding changes in regulation of inflammatory genes of chondrocytes and intraarticular macrophages.^57^ Liraglutide also displayed further anti‐catabolic effects by decreasing the activity of MMPs.^57^

However, outcomes in initial prospective in clinical studies are modest. In a RCT of patients ages 18–74 years‐old with a BMI ≥ 28 kg/m^2^ and with knee OA, patients underwent 8 weeks of dietary intervention for weight loss, followed by randomization to 52‐weeks of either treatment with liraglutide or placebo.^58^ Outcomes measured in this study included body weight changes and the Knee Osteoarthritis Outcome Score (KOOS). The results showed a significant weight loss in the liraglutide group although no change in KOOS at the 52‐week endpoint. In contrast, another RCT investigated the effects of liraglutide on weight loss at one‐year. The results showed that patients self‐reported improvements in physical functioning by improved KOOS, but did not attain significant change in physical activity level.^59^

The efficacy of other GLP1a, including semaglutide, and dual agonists of GLP‐1 and glucose‐dependent insulinotropic polypeptide (GIP), such as tirzepatide, as a pharmacotherapy for OA are unknown. Pertinently, a multi‐center phase 3 clinical trial studying the effect of once weekly semaglutide injections in patients with obesity and knee OA has recently concluded, with pertinent outcomes including changes in physical function, joint stiffness, and WOMAC pain score.^60^ The investigation into the role of GLP‐1a and dual agonists of GLP‐1 in the pathophysiology of OA and inflammation is an ongoing subject of interest as these agents become more widely prescribed. The additive potential for GLP‐1a medications to function as both a treatment for obesity and OA, their ability to regulate systemic and intraarticular inflammation, and potential to contribute to the disease modification of OA, necessitates future research in this area. Additional research is required to confirm both the effectiveness and the potential for disease‐modifying outcomes of novel pharmacotherapies employed in the treatment of obesity and OA. These pharmacotherapies may serve a dual purpose in managing and alleviating chronic inflammatory disease states.

## 6 CONCLUSION

Once thought to be a disease of “wear and tear,” the interplay between OA and both local and systemic inflammation is crucially related. The symptoms of OA may be further exacerbated by the chronic inflammatory state produced by obesity. The improvement of symptoms related to OA are more closely related to reduction of inflammatory states as opposed to simply weight reduction. A healthy diet and regular exercise remain at the core of treatment recommendations for both weight loss and symptomatic OA. However, with new peptide agonists on the horizon, the value of these treatment options needs to be further investigated. Further research is necessary to assess novel pharmacotherapies used for treating obesity, as they may play a dual role in treating and reducing chronic inflammatory disease states.

## AUTHOR CONTRIBUTIONS

NJ: study design, literature search, generation of figures, writing of the manuscript. WH: study design, literature search, generation of figures, writing of the manuscript. LS: study design, writing of the manuscript. SK: study design, writing of the manuscript.

## CONFLICT OF INTEREST STATEMENT

No conflict of interest was declared.

## References

[1] Hunter DJ , Schofield D , Callander E . The individual and socioeconomic impact of osteoarthritis. Nat Rev Rheumatol. 2014;10(7):437‐441. doi:10.1038/nrrheum.2014.44 24662640

[2] Plotnikoff R , Karunamuni N , Lytvyak E , et al. Osteoarthritis prevalence and modifiable factors: a population study. BMC Public Health. 2015;15:1195. doi:10.1186/s12889-015-2529-0 26619838 PMC4666016

[3] Fasshauer M , Blüher M . Adipokines in health and disease. Trends Pharmacol Sci. 2015;36(7):461‐470. doi:10.1016/j.tips.2015.04.014 26022934

[4] Pearson MJ , Herndler‐Brandstetter D , Tariq MA , et al. IL‐6 secretion in osteoarthritis patients is mediated by chondrocyte‐synovial fibroblast cross‐talk and is enhanced by obesity. Sci Rep. 2017;7(1):3451. doi:10.1038/s41598-017-03759-w 28615667 PMC5471184

[5] Reddy P , Lent‐Schochet D , Ramakrishnan N , McLaughlin M , Jialal I . Metabolic syndrome is an inflammatory disorder: a conspiracy between adipose tissue and phagocytes. Clin Chim Acta. 2019;496:35‐44. doi:10.1016/j.cca.2019.06.019 31229566

[6] Koonce RC , Bravman JT . Obesity and osteoarthritis: more than just wear and tear. J Am Acad Orthop Surg. 2013;21(3):161‐169. doi:10.5435/jaaos-21-03-161 23457066

[7] O'Reilly S , Doherty M . Lifestyle changes in the management of osteoarthritis. Best Pract Res Clin Rheumatol. 2001;15(4):559‐568. doi:10.1053/berh.2001.0173 11567539

[8] Maciejewski ML , Arterburn DE , Van Scoyoc L , et al. Bariatric surgery and long‐term durability of weight loss. JAMA Surg. 2016;151(11):1046‐1055. doi:10.1001/jamasurg.2016.2317 27579793 PMC5112115

[9] Peterli R , Steinert RE , Woelnerhanssen B , et al. Metabolic and hormonal changes after laparoscopic roux‐en‐Y gastric bypass and sleeve gastrectomy: a randomized, prospective trial. Obes Surg. 2012;22(5):740‐748. doi:10.1007/s11695-012-0622-3 22354457 PMC3319900

[10] Martinou E , Stefanova I , Iosif E , Angelidi AM . Neurohormonal changes in the gut‐brain Axis and underlying neuroendocrine mechanisms following bariatric surgery. Int J Mol Sci. 2022;23(6):1‐40. doi:10.3390/ijms23063339 PMC8954280 35328759

[11] Sarma S , Palcu P . Weight loss between glucagon‐like peptide‐1 receptor agonists and bariatric surgery in adults with obesity: a systematic review and meta‐analysis. Obesity (Silver Spring). 2022;30(11):2111‐2121. doi:10.1002/oby.23563 36321278

[12] Srivastava AK , Surgical Management of Osteoarthritis of the knee work group SaotAAoOS . American Academy of Orthopaedic surgeons clinical practice guideline summary of surgical Management of Osteoarthritis of the knee. J Am Acad Orthop Surg. 2023;31(24):1211‐1220. doi:10.5435/jaaos-d-23-00338 37883429

[13] Robson EK , Hodder RK , Kamper SJ , et al. Effectiveness of weight‐loss interventions for reducing pain and disability in people with common musculoskeletal disorders: a systematic review with meta‐analysis. J Orthop Sports Phys Ther. 2020;50(6):319‐333. doi:10.2519/jospt.2020.9041 32272032

[14] Salis Z , Sainsbury A , I Keen H , Gallego B , Jin X . Weight loss is associated with reduced risk of knee and hip replacement: a survival analysis using osteoarthritis initiative data. Int J Obes (Lond). 2022;46(4):874‐884. doi:10.1038/s41366-021-01046-3 35017711

[15] Bartels EM , Henrotin Y , Bliddal H , Centonze P , Henriksen M . Relationship between weight loss in obese knee osteoarthritis patients and serum biomarkers of cartilage breakdown: secondary analyses of a randomised trial. Osteoarthr Cartil. 2017;25(10):1641‐1646. doi:10.1016/j.joca.2017.06.009 28689920

[16] Kawai T , Autieri MV , Scalia R . Adipose tissue inflammation and metabolic dysfunction in obesity. Am J Physiol Cell Physiol. 2021;320(3):C375‐C391. doi:10.1152/ajpcell.00379.2020 33356944 PMC8294624

[17] Rahmati M , Mobasheri A , Mozafari M . Inflammatory mediators in osteoarthritis: a critical review of the state‐of‐the‐art, current prospects, and future challenges. Bone. 2016;85:81‐90. doi:10.1016/j.bone.2016.01.019 26812612

[18] Uribe‐Querol E , Rosales C . Neutrophils actively contribute to obesity‐associated inflammation and pathological complications. Cells. 2022;11(12):1‐27. doi:10.3390/cells11121883 PMC9221045 35741012

[19] Herishanu Y , Rogowski O , Polliack A , Marilus R . Leukocytosis in obese individuals: possible link in patients with unexplained persistent neutrophilia. Eur J Haematol. 2006;76(6):516‐520. doi:10.1111/j.1600-0609.2006.00658.x 16696775

[20] Wang G , Jing W , Bi Y , et al. Neutrophil elastase induces chondrocyte apoptosis and facilitates the occurrence of osteoarthritis. Front Pharmacol. 2021;12:666162. doi:10.3389/fphar.2021.666162 33935789 PMC8080035

[21] Hsueh MF , Zhang X , Wellman SS , Bolognesi MP , Kraus VB . Synergistic roles of macrophages and neutrophils in osteoarthritis progression. Arthritis Rheumatol. 2021;73(1):89‐99. doi:10.1002/art.41486 32783329 PMC7876152

[22] Lumeng CN , Bodzin JL , Saltiel AR . Obesity induces a phenotypic switch in adipose tissue macrophage polarization. J Clin Invest. 2007;117(1):175‐184. doi:10.1172/jci29881 17200717 PMC1716210

[23] Winer DA , Winer S , Shen L , et al. B cells promote insulin resistance through modulation of T cells and production of pathogenic IgG antibodies. Nat Med. 2011;17(5):610‐617. doi:10.1038/nm.2353 21499269 PMC3270885

[24] Burt KG , Scanzello CR . B cells in osteoarthritis: simply a sign or a target for therapy? Osteoarthr Cartil. 2023;31(9):1148‐1151. doi:10.1016/j.joca.2023.06.002 PMC10680778 37328048

[25] Xie X , van Delft MAM , Shuweihdi F , et al. Auto‐antibodies to post‐translationally modified proteins in osteoarthritis. Osteoarthr Cartil. 2021;29(6):924‐933. doi:10.1016/j.joca.2021.03.008 33757859

[26] Park CS , Shastri N . The role of T cells in obesity‐associated inflammation and metabolic disease. Immune Netw. 2022;22(1):e13. doi:10.4110/in.2022.22.e13 35291655 PMC8901709

[27] Scanzello CR , McKeon B , Swaim BH , et al. Synovial inflammation in patients undergoing arthroscopic meniscectomy: molecular characterization and relationship to symptoms. Arthritis Rheum. 2011;63(2):391‐400. doi:10.1002/art.30137 21279996 PMC3260472

[28] Huang X , Liu G , Guo J , Su Z . The PI3K/AKT pathway in obesity and type 2 diabetes. Int J Biol Sci. 2018;14(11):1483‐1496. doi:10.7150/ijbs.27173 30263000 PMC6158718

[29] Sun K , Luo J , Guo J , Yao X , Jing X , Guo F . The PI3K/AKT/mTOR signaling pathway in osteoarthritis: a narrative review. Osteoarthr Cartil. 2020;28(4):400‐409. doi:10.1016/j.joca.2020.02.027 32081707

[30] Bartok B , Boyle DL , Liu Y , et al. PI3 kinase δ is a key regulator of synoviocyte function in rheumatoid arthritis. Am J Pathol. 2012;180(5):1906‐1916. doi:10.1016/j.ajpath.2012.01.030 22433439

[31] Lin C , Liu L , Zeng C , et al. Activation of mTORC1 in subchondral bone preosteoblasts promotes osteoarthritis by stimulating bone sclerosis and secretion of CXCL12. Bone Res. 2019;7:5. doi:10.1038/s41413-018-0041-8 30792936 PMC6381187

[32] Mutabaruka MS , Aoulad Aissa M , Delalandre A , Lavigne M , Lajeunesse D . Local leptin production in osteoarthritis subchondral osteoblasts may be responsible for their abnormal phenotypic expression. Arthritis Res Ther. 2010;12(1):R20. doi:10.1186/ar2925 20141628 PMC2875652

[33] Bao JP , Chen WP , Feng J , Hu PF , Shi ZL , Wu LD . Leptin plays a catabolic role on articular cartilage. Mol Biol Rep. 2010;37(7):3265‐3272. doi:10.1007/s11033-009-9911-x 19876764

[34] Buettner C , Muse ED , Cheng A , et al. Leptin controls adipose tissue lipogenesis via central, STAT3‐independent mechanisms. Nat Med. 2008;14(6):667‐675. doi:10.1038/nm1775 18516053 PMC2671848

[35] Koskinen A , Juslin S , Nieminen R , Moilanen T , Vuolteenaho K , Moilanen E . Adiponectin associates with markers of cartilage degradation in osteoarthritis and induces production of proinflammatory and catabolic factors through mitogen‐activated protein kinase pathways. Arthritis Res Ther. 2011;13(6):R184. doi:10.1186/ar3512 22077999 PMC3334633

[36] Bokarewa M , Nagaev I , Dahlberg L , Smith U , Tarkowski A . Resistin, an adipokine with potent proinflammatory properties. J Immunol. 2005;174(9):5789‐5795. doi:10.4049/jimmunol.174.9.5789 15843582

[37] Perruccio AV , Mahomed NN , Chandran V , Gandhi R . Plasma adipokine levels and their association with overall burden of painful joints among individuals with hip and knee osteoarthritis. J Rheumatol. 2014;41(2):334‐337. doi:10.3899/jrheum.130709 24334649

[38] Staikos C , Ververidis A , Drosos G , Manolopoulos VG , Verettas DA , Tavridou A . The association of adipokine levels in plasma and synovial fluid with the severity of knee osteoarthritis. Rheumatology (Oxford). 2013;52(6):1077‐1083. doi:10.1093/rheumatology/kes422 23382357

[39] Iwata M , Ochi H , Hara Y , et al. Initial responses of articular tissues in a murine high‐fat diet‐induced osteoarthritis model: pivotal role of the IPFP as a cytokine fountain. PLoS One. 2013;8(4):e60706. doi:10.1371/journal.pone.0060706 23593289 PMC3625196

[40] Ioan‐Facsinay A , Kloppenburg M . An emerging player in knee osteoarthritis: the infrapatellar fat pad. Arthritis Res Ther. 2013;15(6):225. doi:10.1186/ar4422 24367915 PMC3979009

[41] Klein‐Wieringa IR , Kloppenburg M , Bastiaansen‐Jenniskens YM , et al. The infrapatellar fat pad of patients with osteoarthritis has an inflammatory phenotype. Ann Rheum Dis. 2011;70(5):851‐857. doi:10.1136/ard.2010.140046 21242232

[42] Gross JB , Guillaume C , Gegout‐Pottie P , et al. The infrapatellar fat pad induces inflammatory and degradative effects in articular cells but not through leptin or adiponectin. Clin Exp Rheumatol. 2017;35(1):53‐60. 27908299

[43] Eymard F , Pigenet A , Citadelle D , et al. Induction of an inflammatory and prodegradative phenotype in autologous fibroblast‐like synoviocytes by the infrapatellar fat pad from patients with knee osteoarthritis. Arthritis Rheumatol. 2014;66(8):2165‐2174. doi:10.1002/art.38657 24719336

[44] Han W , Aitken D , Zhu Z , et al. Hypointense signals in the infrapatellar fat pad assessed by magnetic resonance imaging are associated with knee symptoms and structure in older adults: a cohort study. Arthritis Res Ther. 2016;18(1):234. doi:10.1186/s13075-016-1130-y 27729069 PMC5059934

[45] Miller RE , Miller RJ , Malfait AM . Osteoarthritis joint pain: the cytokine connection. Cytokine. 2014;70(2):185‐193. doi:10.1016/j.cyto.2014.06.019 25066335 PMC4254338

[46] Hong N , Lin Y , Ye Z , et al. The relationship between dyslipidemia and inflammation among adults in east coast China: a cross‐sectional study. Front Immunol. 2022;13:937201. doi:10.3389/fimmu.2022.937201 36032093 PMC9403313

[47] Gierman LM , Kühnast S , Koudijs A , et al. Osteoarthritis development is induced by increased dietary cholesterol and can be inhibited by atorvastatin in APOE*3Leiden.CETP mice–a translational model for atherosclerosis. Ann Rheum Dis. 2014;73(5):921‐927. doi:10.1136/annrheumdis-2013-203248 23625977

[48] Dickson BM , Roelofs AJ , Rochford JJ , Wilson HM , De Bari C . The burden of metabolic syndrome on osteoarthritic joints. Arthritis Res Ther. 2019;21(1):289. doi:10.1186/s13075-019-2081-x 31842972 PMC6915944

[49] Sandoval DA , D'Alessio DA . Physiology of proglucagon peptides: role of glucagon and GLP‐1 in health and disease. Physiol Rev. 2015;95(2):513‐548. doi:10.1152/physrev.00013.2014 25834231

[50] Watanabe JH , Kwon J , Nan B , Reikes A . Trends in glucagon‐like peptide 1 receptor agonist use, 2014 to 2022. J Am Pharm Assoc (2003). 2023;64(1):133‐138. doi:10.1016/j.japh.2023.10.002 37821008

[51] Cornu M , Modi H , Kawamori D , Kulkarni RN , Joffraud M , Thorens B . Glucagon‐like peptide‐1 increases beta‐cell glucose competence and proliferation by translational induction of insulin‐like growth factor‐1 receptor expression. J Biol Chem. 2010;285(14):10538‐10545. doi:10.1074/jbc.M109.091116 20145256 PMC2856261

[52] Sandsdal RM , Juhl CR , Jensen SBK , et al. Combination of exercise and GLP‐1 receptor agonist treatment reduces severity of metabolic syndrome, abdominal obesity, and inflammation: a randomized controlled trial. Cardiovasc Diabetol. 2023;22(1):41. doi:10.1186/s12933-023-01765-z 36841762 PMC9960425

[53] Chen J , Xie JJ , Shi KS , et al. Glucagon‐like peptide‐1 receptor regulates endoplasmic reticulum stress‐induced apoptosis and the associated inflammatory response in chondrocytes and the progression of osteoarthritis in rat. Cell Death Dis. 2018;9(2):212. doi:10.1038/s41419-017-0217-y 29434185 PMC5833344

[54] Miao XY , Gu ZY , Liu P , et al. The human glucagon‐like peptide‐1 analogue liraglutide regulates pancreatic beta‐cell proliferation and apoptosis via an AMPK/mTOR/P70S6K signaling pathway. Peptides. 2013;39:71‐79. doi:10.1016/j.peptides.2012.10.006 23116613

[55] Que Q , Guo X , Zhan L , et al. The GLP‐1 agonist, liraglutide, ameliorates inflammation through the activation of the PKA/CREB pathway in a rat model of knee osteoarthritis. J Inflamm (Lond). 2019;16:13. doi:10.1186/s12950-019-0218-y 31182934 PMC6554939

[56] Zhang W , Robertson WB , Zhao J , Chen W , Xu J . Emerging trend in the pharmacotherapy of osteoarthritis. Front Endocrinol (Lausanne). 2019;10:431. doi:10.3389/fendo.2019.00431 31312184 PMC6614338

[57] Meurot C , Martin C , Sudre L , et al. Liraglutide, a glucagon‐like peptide 1 receptor agonist, exerts analgesic, anti‐inflammatory and anti‐degradative actions in osteoarthritis. Sci Rep. 2022;12(1):1567. doi:10.1038/s41598-022-05323-7 35091584 PMC8799666

[58] Gudbergsen H , Overgaard A , Henriksen M , et al. Liraglutide after diet‐induced weight loss for pain and weight control in knee osteoarthritis: a randomized controlled trial. Am J Clin Nutr. 2021;113(2):314‐323. doi:10.1093/ajcn/nqaa328 33471039

[59] Bartholdy C , Overgaard A , Gudbergsen H , Bliddal H , Kristensen LE , Henriksen M . Changes in physical activity during a one‐year weight loss trial with liraglutide vs placebo in participants with knee osteoarthritis: secondary analyses of a randomised controlled trial. Osteoarthr Cartil Open. 2022;4(2):100255. doi:10.1016/j.ocarto.2022.100255 36475294 PMC9718081

[60] Research Study Looking at How Well Semaglutide Works in People Suffering From Obesity and Knee Osteoarthritis . ClinicalTrials.Gov identifier: NCT050647352024.

